# Isolation and Molecular Characterization of Thirteen R2R3-MYB Transcription Factors from *Epimedium sagittatum*

**DOI:** 10.3390/ijms14010594

**Published:** 2012-12-27

**Authors:** Wenjun Huang, Wei Sun, Haiyan Lv, Gong Xiao, Shaohua Zeng, Ying Wang

**Affiliations:** 1 Key Laboratory of Plant Germplasm Enhancement and Specialty Agriculture, Wuhan Botanical Garden, Chinese Academy of Sciences, Wuhan 430074, Hubei, China; E-Mails: wjhuang@wbgcas.cn (W.H.); hyl@wbgcas.cn (H.L.); gongxiaobio@gmail.com (G.X.); 2 Key Laboratory of Plant Resources Conservation and Sustainable Utilization, South China Botanical Garden, Chinese Academy of Sciences, Guangzhou 510650, Guangdong, China; E-Mails: djsunwei@gmail.com (W.S.); shhzeng@scbg.ac.cn (S.Z.)

**Keywords:** *Epimedium*, medicinal plant, flavonoid pathway, MYB, transcription factor

## Abstract

*Epimedium sagittatum* (Sieb. et Zucc.) Maxim, a popular traditional Chinese medicinal plant, has been widely used for treating sexual dysfunction and osteoporosis in China. The main bioactive components in herba epimedii are prenylated flavonol glycosides, which are end products of a branch of the flavonoid biosynthetic pathway. The MYB transcription factors (TF) act as activators or repressors to regulate the flavonoid pathway. In this study, 13 full-length cDNA clones of *R2R3-MYB* TFs from *E. sagittatum* (designated as *EsMYB1* to *EsMYB13)* were isolated and characterized. Sequence similarity and phylogenetic analysis placed nine *R2R3-MYB* members of *E. sagittatum* into five subgroups of the *Arabidopsis R2R3-MYB* family, while four members were not clustered into a defined subgroup. The number and length of introns from Epimedium *R2R3-MYB* genes varied significantly, but intron positions and phases were well conserved. Expression patterns of Epimedium *R2R3-MYB* genes in various tissues showed diverse. Finally, it is suggested that five Epimedium *R2R3-MYB* genes may be involved in regulating the flavonoid pathway and could be used as valuable candidate genes for metabolic engineering studies in future. Sequence information of 13 *R2R3-MYB* genes discovered here will also provide an entry point into the overview of whole *R2R3-MYB* family in Epimedium.

## 1. Introduction

Transcription factors (TFs) play a central role in developmental and metabolic programs by regulating the transcription expression of downstream target genes. The MYB proteins comprise one of the largest TF families in the plant kingdom [1,2]. MYB proteins have two distinct regions, an *N*-terminal conserved MYB DNA-binding domain (MYB domain) and a diverse *C*-terminal modulator region that is responsible for the regulatory activity of the protein. The MYB domain is highly conserved among plants, yeast and animals [3], and its consensus sequence contains approximately 50 amino acid residues with regularly spaced tryptophan forming a helix-turn-helix structure [4]. Based on the number of MYB domain, the MYB family can be divided into four classes, 1R-, R2R3-, 3R- and 4R-MYB proteins [2,5]. *R2R3-MYB* proteins are specific to plants and are also the most abundant type in plants, with greater than 100 *R2R3-MYB* members in the genomes of dicots and monocots [5–8]. Plant *R2R3-MYB* proteins play an important role in many biological processes including primary and secondary metabolism, cell fate and identity, developmental processes and responses to biotic and abiotic stress [5,9,10].

Since the first gene (*C1*) encoding a plant MYB protein was identified in maize [11], a tremendous number of plant MYB genes have been characterized, of which many *R2R3-MYB* genes were demonstrated to regulate the phenylpropanoid and flavonoid biosynthetic pathway in several plant species, including *Arabidopsis*, apple, grapevine, maize, petunia and snapdragon [12]. In *Arabidopsis* for example, *AtTT2* regulates proanthocyanin (PA) biosynthesis in the seed coat [13], while *AtPAP1* and *AtPAP2* activate the phenylpropanoid biosynthetic genes and enhance anthocyanin accumulation in vegetative tissues [14]. In addition, grapevine *VvMYBPA1*, *VvMYBA1*, *VvMYB5a* and *VvMYBF1* controls PA, anthocyanin, phenylpropanoid and flavonol biosynthesis, respectively [9,15–17]. It is well established that MYB TFs interact with bHLH TFs, which act together with WD40 protein to regulate the flavonoid biosynthetic pathway and cell fate [18–20].

Herba epimedii, one of the most popular traditional Chinese medicines, is collected from the dried aerial parts of *Epimedium* species (Berberidaceae family), widely distributed in China [21]. Herba epimedii has extensive pharmacological efficacy for treating sexual dysfunction and infertility, preventing cardiovascular diseases, protecting against osteoporosis, strengthening immune system and improving whole body health as well as possessing anti-oxidation, anti-tumor, and anti-aging effects [22,23]. The herb *E. sagittatum* (Sieb. et Zucc.) Maxim recorded in the Chinese Pharmacopoeia [24] contains high level of bioactive compounds and is widely used as a Chinese herbal medicine. In addition, *Epimedium* species are used as ground covers and ornamental plants due to the abundance of colors and patterns in their leaves and flowers [25].

It has been reported that the main constituents of *Epimedium* species which contribute to various bioactivities are prenylated flavonol glycosides [22,26], end-products of a branch of the flavonoid biosynthetic pathway. To date, more than 260 compounds have been isolated and identified in the genus *Epimedium*, among which C-8 prenylated flavonol glycosides are the major compounds, and four of these (epimedin A, B, C and icariin) are used as important bioactive markers for quality control and chemotaxonomic classification [22,27]. However, the molecular biosynthesis and regulatory mechanism to produce these bioactive components is still unclear. The recent development of an EST database from *E. sagittatum* will accelerate the molecular isolation of genes involved in the flavonoid pathway [25].

Wild natural resources of medicinal *Epimedium* species have declined dramatically in recent times due to years of over-harvesting and habitat destruction and these species have now become endangered [28]. Considering the shortage of natural resources and the large growing market demand for natural medicines, it is essential to better understand the biosynthesis and regulation of bioactive compounds in high-quality germplasm resources, in order to enable development of new cultivars with the improved traits for large-scale cultivation.

The *R2R3-MYB* family has been divided into several subgroups [5,29], and the functions of *R2R3-MYB* proteins from the same subgroup are broadly conserved in different angiosperms [2]. Thus, it may be predicted that several MYB proteins could regulate the flavonoid biosynthetic pathway and other secondary metabolite pathways involved in the biosynthesis of bioactive compounds in *E. sagittatum*. However, no data has been available for *R2R3-MYB* genes in the genus *Epimedium* to date. This study also is thus the first to analyze the genomic structure of the *R2R3-MYB* genes in *E. sagittatum* and their phylogenetic relationships with other plant *MYB* genes. In short, we aim to isolate and characterize several members of *R2R3-MYB* family that are proposed to play an important role in controlling the flavonoid biosynthetic pathway to pave the way for genetic manipulation of bioactive compound biosynthesis in *Epimedium* species. Specifically, we isolated and characterized 13 different *R2R3-MYB* full-length sequences from *E. sagittatum*. The full-length coding sequences enabled us to explore their phylogenetic relationships with other plant *MYB* genes and further predict their biological functions. We also analyzed the genomic structures of these isolated *R2R3-MYB* genes to obtain insights into their evolution. The mRNA expression patterns of these *MYB* genes were examined by quantitative RT-PCR (qRT-PCR) assay in various tissues. At last, we found that five *R2R3-MYB* genes are likely to regulate the flavonoid biosynthetic pathway in Epimedium.

## 2. Results

### 2.1. Isolation and Sequence Analysis of 13 *R2R3-MYB* Genes from Epimedium

**Figure 1:**
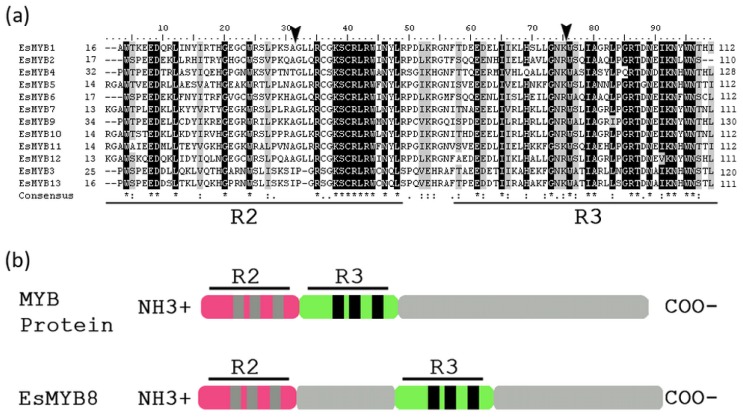
Sequence alignment of 12 *R2R3-MYB* proteins within the MYB domains from *E. sagittatum* except EsMYB8 and their R2 and R3 MYB domains arrangement. (**a**) Identical residues are shown in black and similar residues in gray. The R2 and R3 MYB domains are underlined. The two arrowheads indicate the intron I and intron II insertion site, respectively. (**b**) The R2 and R3 MYB domains are arranged in tandem in 12 of 13 EsMYB proteins, except EsMYB8 protein in which a fragment of about 50 amino acid spaces the R2 and R3 repeat domain.

A total of 13 full-length cDNA clones of *R2R3-MYB*TFs were isolated from leaves of *E. sagittatum* using homology-based cloning, RACE (rapid amplification of cDNA ends) technology and fishing for candidate ESTs encoding MYB proteins in the Epimedium EST database [25]. Each of the *R2R3-MYB* cDNA clones contained one whole open reading frame (ORF), varying from 735 bp (*EsMYB5*) to 1323 bp (*EsMYB2*) in length and encoding 244 to 440 amino acids (aa) with an average length of about 310 aa (Table S1). All Epimedium *R2R3-MYB* sequences contained the R2 and R3 MYB domain identified through conserved domain analysis, placing them in the large *R2R3-MYB* family (Figure 1a). In most of EsMYB members, the R2 and R3 MYB domains were arranged in tandem as is typical for plant *R2R3-MYB* proteins, but a fragment of approximate 50 aa separated the R2 and R3 MYB domain of EsMYB8 (Figure 1b). Similarly to other plant *R2R3-MYB* proteins, all EsMYB proteins were highly conserved in the *N*-terminal region and highly variable in the *C*-terminal region. Alignment of the deduced amino acid sequences for 12 *EsMYB* genes, excluding the atypical *EsMYB8*, revealed an overall protein identity varying from 5% to 66%, while the MYB domain represented a high level of amino acid conservation (44%–90% identities), particularly in the R3 repeat domain (Figure 1a). Five pairs of EsMYB proteins with very high identities (>80%) in their MYB domains were found: EsMYB3 and EsMYB13, EsMYB7 and EsMYB10, EsMYB2 and EsMYB6, EsMYB1 and EsMYB12, and EsMYB5 and EsMYB11 (Figure 1a).

A blast search of all *EsMYB* genes in the *Arabidopsis* nr database revealed that each *EsMYB* gene possessed many corresponding homologues from *Arabidopsis* with high identity (Table S2). For example, *EsMYB7* and *EsMYB10* showed high homology with the *Arabidopsis AtTT2* (*AtMYB123*) gene involved in regulating proanthocyanin (PA) accumulation in the seed coat [13], while *EsMYB1* and *EsMYB12* were highly homologous with the transcriptional repressor *AtMYB4*, which negatively regulates the expression of cinnamate 4-hydroxylase [30] (Table S2). In addition, blast analysis of Epimedium *MYB* genes in the nr database excluding *Arabidopsis* revealed that *EsMYB5* and *EsMYB11* shared approximately 50% identity with *VvMYBPA2* which regulates PA biosynthesis in grape [31], and *EsMYB9* shared 56% identity with grape *VvMYB5b* which regulates the flavonoid pathway during fruit development [9] (Table S3), and *EsMYB8* was highly similar to *AtMYB* (At5g58900) and other plant *DIV* (*DIVARICATA*) and *DIV*-like genes (e.g., *AmDIV*, *BlDIV*, *HmDIV* and *ZmDIV*) involved in the dorsoventral asymmetry of flowers [32,33] (Table S3).

To gain further insight into the predicted functions of *MYB* genes from Epimedium, additional sequence analysis was performed. The bHLH interacting motif ([D/E]Lx2[R/K]x3Lx6Lx3R) in the R3 domain [34] was present in five Epimedium *R2R3-MYB* genes, including *EsMYB1*, *EsMYB7*, *EsMYB9*, *EsMYB10* and *EsMYB12* (data not shown). Each *EsMYB* gene clustered into one subgroup of the *Arabidopsis R2R3-MYB* family possessed the subgroup-specific conserved motifs located outside the MYB domain [5,29]. *EsMYB7* and *EsMYB10* shared a similar motif with the *R2R3-MYB* subgroup 5-specific conserved motif (DExWRLxxT) [5], while *EsMYB1* and *EsMYB12* contained two *R2R3-MYB* subgroup 4-specific conserved motifs: C1 (LlsrGIDPxT/SHRxI/L) and C2 (pdLNLD/ELxiG/S), of which the C2 motif forms part of the region involved in transcriptional repression [30]. Unlike other members of subgroup 4, however *EsMYB12* did not contain a Zinc-finger motif (Cx_1–2_Cx_7–12_Cx_2_C) in the *C*-terminus [29], while *EsMYB1* did (Figure S1). Moreover, *EsMYB9* contained the C1 motif of *R2R3-MYB* subgroup 4 [29] as well as the C3 motif (DDxF[S/P]SFL[N/D]SLIN[E/D]) identified previously in *VvMYB5a* and *VvMYB5b* clusters [9,35], but not the C2 motif [30] (Figure S2). Unlike typical plant *R2R3-MYB* genes, *EsMYB8* has longer linkers (about 55 aa) between the R2 and R3 repeat domains and a specific “VASHAQKYF” motif sequence located towards the end of the R3 MYB domain, and another conserved motif followed closely in the *C*-terminus was also found in the EsMYB8 sequence (Figure S3) [36].

### 2.2. Phylogenetic Relationships and Genomic Structure Analysis of Epimedium *R2R3-MYB* Genes

**Figure 2:**
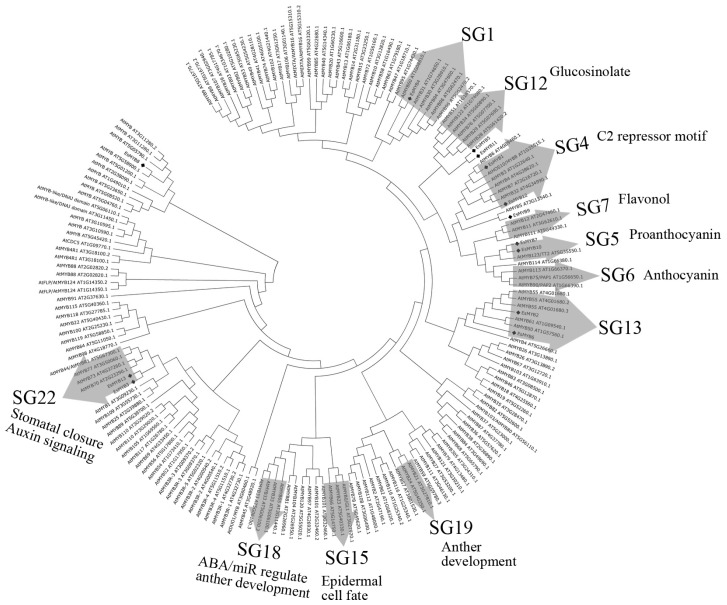
Phylogenetic relationships of 13 *R2R3-MYB* genes from *E. sagittatum* and 166 *MYB* gene models from *Arabidopsis thaliana*. Phylogenetic tree is constructed using the neighbor-joining method by the MEGA 4.0 version [40]. 13 *R2R3-MYB* genes from Epimedium are shown in diamond. All 166 *MYB* gene models are downloaded from the *Arabidopsis thaliana* MYB Transcription Factor database [41] and their gene model accession numbers are shown. *Arabidopsis R2R3-MYB* subgroups are designated as previously reported [5,29]. The putative functions of the different *MYB* genes in the control of secondary metabolite biosynthesis or other biological processes are indicated.

A phylogenetic tree of 13 complete *R2R3-MYB* proteins from *E. sagittatum* and 166 MYB proteins downloaded from the *Arabidopsis* Plant Transcription Factor Database was constructed using the neighbor-joining method. Nine *EsMYB* members clustered into five previously defined subgroups (SG) of the *R2R3-MYB* family in *Arabidopsis*, including SG1, SG4, SG5, SG13 and SG22 [5,29], while four *EsMYB* genes (*EsMYB5*, *EsMYB8*, *EsMYB9* and *EsMYB11*) did not fall into a defined subgroup (Figure 2). In accordance with their SG-specific motifs, *EsMYB1* and *EsMYB12* were clustered into SG4, *EsMYB7* and *EsMYB10* into the SG5; while *EsMYB9* showed a close relationship with *AtMYB5*, but it was not assigned to a defined subgroup (Figure 2). The topology tree indicated that most of EsMYB proteins were placed at the basal position of a group or branch (Figure 2).

Phylogenetic analysis of 13 *R2R3-MYB* genes from *E. sagittatum* with other functionally characterized plant *MYB* genes was also conducted to further predict their potential biological functions. The result was in agreement with that of the phylogenetic tree of Epimedium and *Arabidopsis MYB* sequences (Figure S4). *EsMYB1* showed a close relationship with the *AtMYB4* and *AmMYB308* transcriptional repressors [30,37], while *EsMYB12* was clustered closely with the *FaMYB1* repressor from *Fragaria* x *ananassa* with a high bootstrap value [38] (Figure S4). *EsMYB9* clustered into the *VvMYB5a* and *VvMYB5b* group identified previously [9,35], and *EsMYB7* and *EsMYB10* were clustered with *TT2* homologues from *Arabidopsis* and *Lotus*, but the bootstrap value was relatively low (Figure S4) [13,39]. *EsMYB8* clustered with *DIV* homologues from *Aquilegia coerulea* with a high bootstrap value (Figure S4).

**Figure 3:**
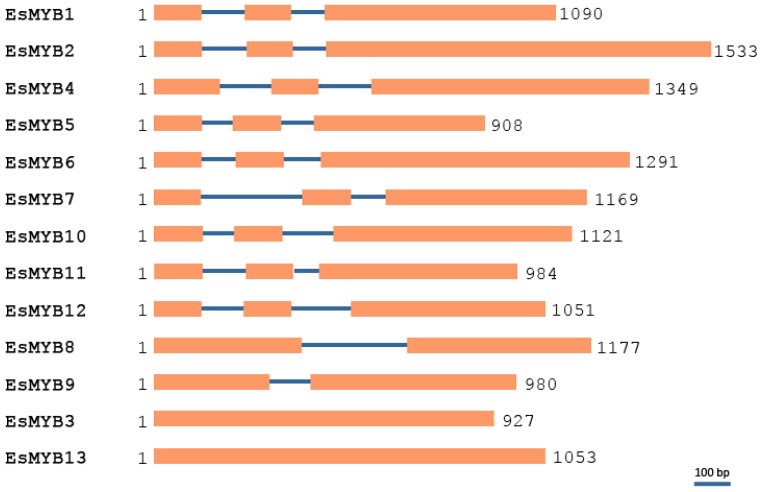
Genomic structures of 13 *R2R3-MYB* genes from *E. sagittatum*. Exons and introns are shown in boxes and lines, respectively. The numbers at the left and right side indicate the position of the translation start codon and stop codon, respectively. The scale represents 100 bp length in nucleotide. Detailed information about exon and intron length and splice junction site is seen in Table S1.

The genomic structure of Epimedium *R2R3-MYB* genes was analyzed by pair-wise alignment of their full-length coding sequences and corresponding genomic DNA (gDNA) sequences. Three types of exon/intron structure were found in the all 13 *R2R3-MYB* genes and designated as Class I, II and III. Class I contained three exons and two introns (nine members); Class II, two exons and one intron (*EsMYB8* and *EsMYB9*); Class III, one exon and no intron (*EsMYB3* and *EsMYB13*) (Figure 3). To further verify the Class III genomic structure and exclude possible DNA contamination in the cDNA isolation, both the full-length cDNA and gDNA clones of *EsMYB3* and *EsMYB13* from cDNA template digested with DNase I and genomic DNA template were amplified and compared at the same time. The band size was as same as expected between the cDNA and gDNA clone of each gene (Figure S5). The average length of both intron I and intron II was found to be same (125 bp), but ranging from 83 bp (*EsMYB10*) to 272 bp (*EsMYB7*) for intron I, and from 66 (*EsMYB11*) to 280 bp (*EsMYB8*) for intron II, respectively (Table S1). The splice junction sites of all *MYB* genes were conserved for GT and AG dinucleotide, except for *EsMYB1* intron II where the acceptor site was TG (Table S1). Moreover, the intron positions of 13 *R2R3-MYB* genes exception *EsMYB8* were conserved, which intron I and intron II inserted between the conserved A and G residues of the R2 MYB domain and (R/K)W residues of the R3 MYB domain, respectively (Figure 1a). The intron II insertion site of *EsMYB8* was between K_134_ and L_135_, which is consistent with the insertion positions and phases from other plant *DIV* genes (Figure S3) [36].

### 2.3. Expression Patterns of Epimedium *R2R3-MYB* Genes in Various Tissues

**Figure 4:**
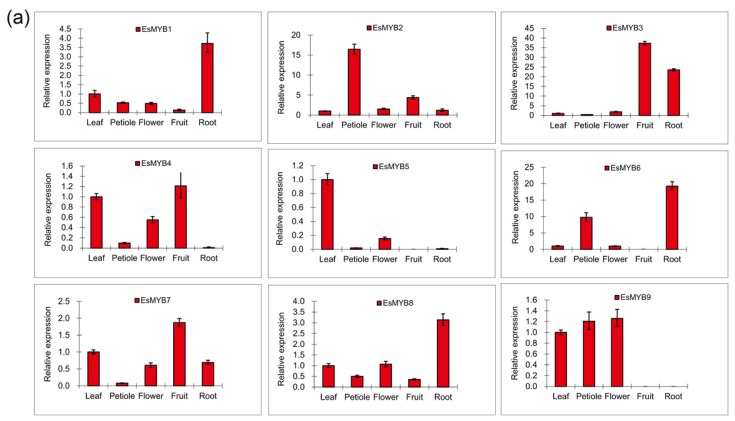

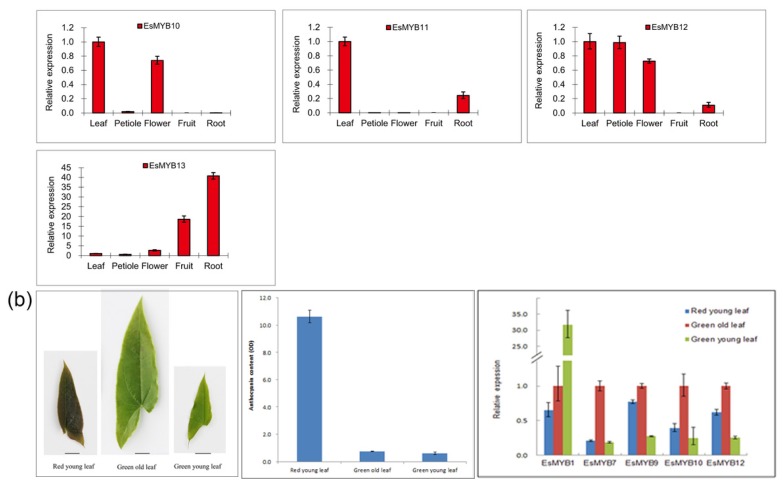
Expression patterns of 13 *R2R3-MYB* genes from *E. sagittatum* in various tissues. (**a**) The mRNA expression patterns of 13 Epimedium *R2R3-MYB* genes in leaf, petiole, flower, fruit and root tissues are examined by qPCR assay. The comparative Ct method is used to determine the relative expression, and the expression level of gene in leaf tissue is set to “1”. (**b**) Five Epimedium *R2R3-MYB* genes (*EsMYB1*, *EsMYB7*, *EsMYB9*, *EsMYB10* and *EsMYB12*) are selected to determine their mRNA expression levels by qPCR assay (right) in the red and green leaf tissues (left) which accumulate different amount of anthocyanin (middle). Three samples are used from two plants for qPCR assay, and the red young leaf and green old leaf are collected from the same plantlet at differential developmental stages, while the green young leaf is collected from another plantlet at the same developmental stage of the red young leaf. Bar = 1 cm. Total anthocyanin content is determined using spectrophotometric method, and the column represents the mean value and SD (standard deviation) indicating from three replicates. The comparative Ct method is used to determine the relative expression level, and the expression level of gene in green old leaf is set to “1”.

Expression patterns of 13 cloned *R2R3-MYB* genes from *E. sagittatum* were investigated by quantitative RT-PCR (qRT-PCR) in various tissues, including young leaf, young petiole, flower, fruit and root (Figure 4a). The results showed that seven *EsMYB* genes were expressed in all tissues tested, with highest abundance in different tissues for each gene, and some *EsMYB* genes were expressed preferentially in the specific tissues, with highest expression in root for *EsMYB1*, *EsMYB6*, *EsMYB8* and *EsMYB13*. In addition, six *EsMYB* genes showed low or no transcripts in fruit tissue, and five *EsMYB* genes were most highly expressed in the young leaf (Figure 4a). Specifically, the mRNA level of *EsMYB9* was highest in the flower, and absent in fruit and root tissues. *EsMYB1* and *EsMYB12* showed different expression patterns, with *EsMYB1* expressed in all tissues and most highly in the root, while *EsMYB12* was expressed in leaf, petiole and flower tissues at the similar level. *EsMYB7* was most abundantly expressed in the fruit and detected in all tissues, while *EsMYB10* was abundantly expressed in leaf and flower tissues, but lowly in fruit and root tissues (Figure 4a).

In order to infer the functions of some *EsMYB* genes further, the mRNA abundances of five selected *EsMYB* (*EsMYB1*, *EsMYB7*, *EsMYB9*, *EsMYB10* and *EsMYB12*) were compared by qRT-PCR in red versus green leaves, which accumulate different anthocyanin contents (Figure 4b). A total of three leaves samples were collected for qRT-PCR analysis, with sample 1 and 2 corresponding to the red young leaf and green old leaf from the same plantlet but collected at different developmental stages, and sample 3 corresponding to the green young leaf collected from another plantlet at the same developmental stage of the sample 1. The results indicated that the expression levels of all five *EsMYB* genes were higher in the green old leaf (sample 2) than in the red young leaf (sample 1) from the same plant, and *EsMYB9*, *EsMYB10* and *EsMYB12* genes were more abundantly expressed in the red young leaf (sample 1) than in the green young leaf (sample 3). *EsMYB1*, on the other hand, was approximately 48 times higher in the green young leaf (sample 3) than in red young leaf (sample 1) (Figure 4b).

## 3. Discussion

In this study, the complete coding sequences and intron-exon structures of 13 Epimedium genes that share the characteristic features of *R2R3-MYB* family were reported. The phylogenetic relationships of Epimedium *R2R3-MYB* genes with other plant *MYB* genes were examined and their mRNA expression patterns in several tissues were also examined to predict their potential functions.

Our data revealed that the MYB domains of Epimedium *R2R3-MYB* genes are highly conserved, whereas the *C*-terminal regions are highly variable, as shown in earlier studies of other plant *MYB* genes [2]. The conserved motif for interaction with *bHLH* TFs, which is usually present in the R3 repeat domain of *R2R3-MYB* regulators controlling the proanthocyanin (PA) and anthocyanin biosynthetic pathway [34,42], was found in five Epimedium *R2R3-MYB* genes. The *Arabidopsis R2R3-MYB* family has been divided into 22 subgroups, and nine of thirteen Epimedium *R2R3-MYB* genes were dispersed into five subgroups and thus contained the subgroup-specific conserved motifs [5,29].

Amino acid motifs of several subgroups are broadly conserved among different species [8,29,43]. *EsMYB1* and *EsMYB12* located in subgroup 4 (SG4) shared the C2 repressor motif (pdLNLD/ELxiG/S), which plays a key role in transcription repression [30]. Besides the C2 repressor motif, all members of SG4 homologues, including *EsMYB1* and *EsMYB12*, harbored a conserved bHLH interaction motif [34], suggesting *EsMYB1* and *EsMYB12* could interact with *bHLH* TF. The overall structural homology between EsMYB1, EsMYB12 and other C2 repressor-containing MYB proteins from different plant species, and the reports that several of them act as transcriptional repressors of the phenylpropanoid pathway suggest that EsMYB1 and EsMYB12 may function as transcriptional repressors of the flavonoid pathway in Epimedium. It is noticeable that *EsMYB1* shared a Zn-finger motif with most of members of SG4, but *EsMYB12* did not. However, it is reported that the strawberry *FaMYB1* which acts as a transcription repressor of the anthocyanin and flavonol pathway also contains the C2 repressor motif but no Zn-finger motif [38]. Moreover, *EsMYB12* showed a closer relationship with *FaMYB1* and had a different mRNA expression pattern with *EsMYB1* which clustered with *AtMYB4* and *AmMYB308*, suggesting divergent tissue-specific activity of these genes.

It is proposed that *MYBs* generally regulate only one branch of the flavonoid pathway [12]. *MYB* regulators of PA biosynthesis have been identified in several plant species, including *AtTT2* from *Arabidopsis*, *LjTT2* from *Lotus japonicus*, *VvMYBPA1* and *VvMYBPA2* from *Vitis vinifera* [13,15,31,39]. *EsMYB7* and *EsMYB10* had high identity with *AtTT2* homologues and were clustered into SG5, and showed close relationships with *MYB* regulators controlling the PA biosynthesis. Although neither protein contained the conserved motif [V(I/V)R(T/P)(K/R)A(I/L/V)(R/K)C] present in the highly variable *C*-terminal region of both *AtTT2* and *OsMYB3* [5,13], this 9-residue consensus sequence was also not found in the amino acid sequence of *LjTT2* controlling PA biosynthesis in *Lotus* [39]. The MYB domains of *EsMYB7* and *EsMYB10* shared greatest identity with *GhMYB10* from *Gossypium hirsutum* and a high similarity of 81%–87% with the *LjTT2* genes. Thus, it is predicted here that *EsMYB7* and *EsMYB10* are possibly the *MYB* regulators of PA biosynthesis in Epimedium. On the other hand, *EsMYB5* and *EsMYB11* showed similarity with *VvMYBPA1*, but they were not clustered together and lacked the *bHLH* interaction motif, which is necessary for the *MYB* regulators of PA biosynthesis. Thus, it is inferred that *EsMYB5* and *EsMYB11* are not true candidate regulators of PA biosynthesis in Epimedium.

Sequence similarity of MYB proteins is mainly confined to the R2 and R3 MYB domains, however MYB proteins can share conserved motifs in their *C*-terminal regions and this may indicate similarities in function [5]. *EsMYB9* showed high identity with *VvMYB5a* and *VvMYB5b* genes of grape and fell into a small clade with five other *MYB* genes involved in the control of various physiological and developmental processes [9,35]. Like the *VvMYB5a* and *VvMYB5b* cluster, *EsMYB9* contained the C1 motif initially described in the *R2R3-MYB* SG4 proteins and the C3 motif only present in the all members of the *VvMYB5a* and *VvMYB5b* small cluster. This, and the fact that transgenic tobacco lines overexpressing both *EsMYB9* and the *VvMYB5a* and *VvMYB5b* genes showed similar phenotypes [9,35] indicates functional conservation of these orthologs in different species. Specifically, for *EsMYB9* overexpression transgenic tobacco lines, significant changes in coloring were showed in petals of transgenic flowers and in stamens where the amount of pigments was clearly greater (Figure S6). In the vegetative tissues of transgenic tobacco lines no visible phenotypic differences were observed, compared to wild-type plants (data not showed).

Exon/intron structures are highly conserved between *R2R3-MYB* family members in Epimedium and several model species. Four groups of *R2R3-MYB* family have been classified in the grape and *Arabidopsis* based on the split position of the R2 and R3 repeats within the MYB domain [8]. Three groups are found in the Epimedium *R2R3-MYB* members, just lacking the group II that only has the R2 repeat split between exon 1 and 2. The fact that just over a dozen Epimedium MYB sequences are available for analysis may provide a reliable explanation. In nine of thirteen Epimedium *R2R3-MYB* genes, exon 1 and 2 encode for almost the entire R2 and R3 MYB domain, and are restricted in length (about 130 bp), as reported in *Arabidopsis* and grape [8]. In addition, exon 3 of *EsMYB* genes encodes for the last region of the R3 repeat and for the *C*-terminal region of the protein, and is the most diverse in size. Nine of thirteen (69%) of Epimedium *R2R3-MYB* genes consisted of 3 exons and 2 introns, which is consistent with the reports that a majority of *Arabidopsis* (59%) and rice (53%) *MYB* genes had this exon/intron structure [6]. It is noticeable that the complete *EsMYB3* and *EsMYB13* proteins were encoded by a single exon, which has not been seen to date in grape [8]. Although the size of introns varied greatly for different *EsMYB* genes, the intron positions and phases were well conserved. *EsMYB8* had only one intron, which split the R3 repeat domain at a distinctive position, but it was highly conserved with the closely related *DIVARICATA* (*DIV*) genes from other plants [36]. Although the sequence information of 13 Epimedium *R2R3-MYB* genes is limited, it may provide an insight into the genomic structure of whole Epimedium *R2R3-MYB* family.

Different *R2R3-MYB* genes from Epimedium had different expression patterns. *EsMYB1* and *EsMYB12* are suggested here to act as transcriptional repressors, and showed different expression profiles. *EsMYB1*was most highly expressed in roots, which is similar to that seen for *EgMYB1* of *Eucalyptus gunnii* preferentially expressed in differentiating secondary xylem of the stems and roots [44]. Moreover, it was observed that *EsMYB1* was higher in the green leaves than in the red leaves which accumulate more anthocyanin contents. The preferential accumulation of *EsMYB1* in lignin-rich tissue suggests a possible role in the regulation of lignin biosynthesis. Unlike *EsMYB1*, *EsMYB12* was abundantly expressed in leaf, petiole and flower tissues which accumulate a certain amount of anthocyanins. *EsMYB12*was not expressed in fruit, which is contrary to the reports about the closely related *FaMYB1* repressor of strawberry. *FaMYB1* showed the highest expression level in the red ripe strawberry fruit and repressed transcription to balance the levels of anthocyanin at the latter stages of fruit maturation [38]. The high expression levels of *EsMYB12* in anthocyanin-rich tissues perhaps suggest that *EsMYB12* is involved in the regulation of anthocyanin biosynthesis. In addition, the fact that *EsMYB12* transcript increased during the maturation stage of leaves and was higher in red young leaves than in green young leaves is counterpart of the *FaMYB1* expression profile [38]. Aside from the transcriptional repressors, *EsMYB9* and both *EsMYB7* and *EsMYB10* are possibly transcriptional activators of the flavonoid and PA biosynthetic pathways, respectively. *EsMYB9*was abundantly expressed in the anthocyanin-rich tissues, which is partly consistent with the high expression of *VvMYB5a* and *VvMYB5b* in leaves and berries of grape [9,35]. Moreover, *EsMYB9* transcript was higher in old leaves than in young leaves, as shown for *VvMYB5b* [9]. Given that *EsMYB9* is suggested to be a *MYB* regulator of the flavonoid pathway, it is surprising that no transcript was found in fruit and roots. The *EsMYB7* expression was not limited to certain tissues, which coincides with the expression patterns of *LjTT2* genes [39]. It is reported that the expression levels of *TT2* homologues correlated with the PA accumulation patterns [13,39]. PA is accumulated in several organs and tissues for *Lotus japonicus*, whereas PA accumulation in *Arabidopsis* is limited to the seed coat, which indicates the different species-specific expression patterns of *TT2* genes [13,39]. The highest expression level of *EsMYB7* was observed in fruit that may accumulate the greatest PA level. *EsMYB10* had high similarity with *EsMYB7*, but it was not expressed in fruit.

## 4. Experimental Section

### 4.1. Plant Materials

Tissues used in this study were sampled in March–April 2011 from the Hunan population of *E. sagittatum* originally transplanted from Hunan province, China in 2006 and grown in the experimental field of Wuhan Botanical Garden, Wuhan, China up to now. Young leaf, young petiole, flower, root and fruit tissues were harvested at the blooming stage except fruits, immediately frozen in liquid nitrogen and stored at −70 °C until required.

### 4.2. DNA and RNA Extraction

Total genomic DNA was extracted from young leaves using the CTAB (cetyltrimethylammonium bromide) method [45]. Total RNA was isolated from fully expanded red leaves using TRIzol reagent (Invitrogen, Carlsbad, CA, USA). The RNA solution was digested with RQ1 RNase-Free DNase (Promega, Madison, WI, USA) to remove any DNA contamination before the reverse transcription reaction.

### 4.3. Reverse Transcription and Isolation of *R2R3-MYB* cDNA Clones

In this study, a total of 13 *R2R3-MYB* cDNA clones were isolated from red young leaves, of which four cDNA clones (designated *EsMYB1* to *EsMYB4*) were obtained using homology-based cloning method as described below, with the remaining nine cDNA clones (designated *EsMYB5* to *EsMYB13*) isolated from the leaf EST database of *E. sagittatum* [25]. Specifically, total RNA (1 μg) from red leaves was reverse transcribed using oligo(dT)_18_ and Superscript II reverse transcriptase (Invitrogen, Carlsbad, CA, USA) and the reaction product was diluted 10-fold for use as cDNA template for MYB clone isolation. Four pairs of degenerate primers for *EsMYB1* to *EsMYB4* (Table S4) were designed to amplify the MYB domain and then the corresponding full-length cDNA clones were amplified with the SMART RACE Amplification kit (Clontech, Mountain View, CA, USA). In other way, the MYB domain was blasted against the *E. sagittatum* EST database and nine *R2R3-MYB* full length cDNA clones were finally obtained using the RACE technology. All gene specific primers for isolation of full-length cDNA clones and the corresponding genomic DNA clones are listed in Table S4. PrimeSTAR HS DNA Polymerase (Takara, Dalian, Liaoning, China) was used to guarantee high fidelity of the sequence. The full-length cDNA sequences of 13 *R2R3-MYB* genes from *E. sagittatum* have been deposited in the GenBank database (accession no. JN426948-JN426960 for *EsMYB1* to *EsMYB13*).

### 4.4. Sequence and Phylogenetic Analysis

Blast program [46] was used to determine candidate genes and ORF finder program [47] was used to search open reading frames (ORF). Spidey program [48] was used to analyze the exon and intron genomic structure and the Conserved Domain program [47] was used to search conserved domains for Epimedium *MYB* genes. Multi-alignment analysis was performed using ClustalW [49] and MEGA 4.0 [40] was used to generate a neighbor-joining phylogenetic tree. Default parameters were used for all programs unless otherwise mentioned.

### 4.5. Quantitative RT-PCR

Quantitative RT-PCR was carried out to detect the expression patterns of 13 isolated *R2R3-MYB* genes in different tissues of *E. sagittatum*. Total RNAs from leaf, petiole, flower and root were extracted with TRIzol reagent (Invitrogen, Carlsbad, CA, USA), while total RNA from fruits was isolated using RNAiso Plus and RNAiso-mate for Plant Tissue kits (Takara, Dalian, Liaoning, China). One microgram of total RNA was reverse transcribed with a Primescript RT reagent kit and gDNA eraser (Takara, Dalian, Liaoning, China) was used to remove any contaminated genomic DNA. The quantitative PCR (qPCR) assay was undertaken according to manufacturer’s instructions using the SYBR Premix Ex Taq II kit (Takara, Dalian, Liaoning, China) and run on an ABI7500 Real-Time PCR machine (Applied Biosystems, Foster, CA, USA). Gene specific primers for qPCR assay were designed using the Primer Premier 5 software and the *Actin* homolog was used as an internal control to normalize the expression level (Table S5). After the end of qPCR program, the melt curve was performed to ensure the amplification of the specific products. The comparative Ct method was used to determine the relative expression [50].

### 4.6. Total Anthocyanin Content Determination

Total anthocyanins were extracted and measured using spectrophotometric method as described by Mancinelli [51]. Red and green leaves were collected at the different developmental stages and frozen at −70 °C until use. The samples (about 200–300 mg) were ground into fine powder with liquid nitrogen. Anthocyanins were extracted with 1% HCl in methanol for 24 h at 4 °C in darkness with occasional shaking. The extracts were centrifuged and decanted carefully, and their absorbance was measured at 530 nm (peak absorption of anthocyanins) and 657 nm (peak absorption of chlorophyll degradation products). The equation A530 − 0.25 × A657 was used to compensate for the absorption of chlorophyll degradation products at 530 nm. Total anthocyanin content was showed as the subtracted absorbance/fresh weight. Three independent replicates were analyzed for each sample.

## 5. Conclusions

In summary, thirteen *R2R3-MYB* genes from *E. sagittatum* were isolated and characterized through homology-based cloning and a systematic survey of EST database followed by full-length sequencing. Detailed sequence information for thirteen *R2R3-MYB* genes provides a forward step into the molecular characterization of whole *R2R3-MYB* family in Epimedium. Based on the results of sequence, phylogenetic and transcription expression analyses it is predicted that five *EsMYB* genes (*EsMYB1*, *EsMYB7*, *EsMYB9*, *EsMYB10* and *EsMYB12*) are likely to be involved in the regulation of various branches of the phenylpropanoid biosynthetic pathway in *E.sagittatum*. The isolation and molecular characterization of *R2R3-MYB* genes will facilitate the understanding of regulation of the flavonoid biosynthetic pathway and even the biosynthesis of the bioactive compounds in Epimedium. The five *EsMYB* genes could be selected as candidate genes for plant metabolic engineering studies in future to produce the desired compounds. Gain-of-function and loss-of-function experiments using transgenic plants are planned to verify the predicted biological functions of the different *EsMYB* genes.

## Supplementary Files

Supplementary File 1 Supplemental Table (PDF, 124 KB)

Supplementary File 2 Supplemental Figures (PDF, 725 KB)
