# Supplementary material for: Isolation and Molecular Characterization of Thirteen R2R3-MYB Transcription Factors from Epimedium sagittatum
Source: Int J Mol Sci. 2012 Dec 27;14(1):594–610. doi: 10.3390/ijms14010594 (PMC3565284; doi:10.3390/ijms14010594)
Supplement: Supplementary File 1 — Supplemental Table (PDF, 124 KB) [file ijms-14-00594-s001.pdf]

Supplementary Information

Table S1. Length of epimedii R2R3-MYB coding sequences and introns with their predicted splice junctions [a].

Gene name	Length (bp) Coding Sequence (bp)	Protein Sequence (aa)	Intron I (bp)	Intron II (bp)	Intron I (phase 1) 5'Splice site	3'Splice site	Intron II (phase 2) 5'Splice site	3'Splice site
EsMYB1	888	295	112	90	GCAG:*GT*ATTC	ATTC*AG*:GT	AACAA:*GT*AAGA	TTTC***TG***:GTGG
EsMYB2	1323	440	121	89	GCAG:*GT*AAGG	TTTC*AG*:GT	AATAG:*GT*ATTA	CACC*AG*:GTGG
EsMYB3	927	308	none	none	none	none	none	none
EsMYB4	1068	355	141	140	ACTG:*GT*GAAA	TTGA*AG*:GA	AACAA:*GT*AATT	GAGC*AG*:ATGG
EsMYB5	735	244	85	88	GCAG:*GT*AAAT	CTTC*AG*:GT	AACAA:*GT*GAGT	TCGC*AG*:GTGG
EsMYB6	1101	366	92	98	GCAG:*GT*AACT	TTGC*AG*:GA	AACAG:*GT*ATTG	TTGT*AG*:GTGG
EsMYB7	804	267	272	93	GCCG:*GT*AAGA	GTGC*AG*:GT	AACAG:*GT*GCTT	GTTT*AG*:ATGG
EsMYB8	897	298	none	280	none	none	CACAA:*GT*AAGT	TTCC*AG*:GCTC
EsMYB9	873	290	none	107	none	none	AACAG:*GT*GATT	TTGC*AG*:ATGG
EsMYB10	903	300	83	135	GCAG:*GT*AAAG	ACGT*AG*:GT	AACAG:*GT*ATTA	TTGT*AG*:ATGG
EsMYB11	801	266	117	66	GCAG:*GT*AAAT	TTTC*AG*:GT	AGCAA:*GT*GAGT	TTAC*AG*:GTGG
EsMYB12	783	260	109	159	GCAG:*GT*TGGT	CCTC*AG*:GT	AACAG:*GT*GCGA	TTAT*AG*:GTGG
EsMYB13	1053	350	none	none	none	none	none	none

[a] Coding sequences indicate the length in nucleotides from the translation start codon to the stop codon and protein sequences correspond to the deduced amino acid length of coding sequence. Intron I and II represent the first and second introns, respectively. Intron phase refers to the position in a codon where the intron is inserted: after the first nucleotide (phase 1) or after the second nucleotide (phase 2) of a codon. Italic nucleotide pairs GT and AG (except *EsMYB1* intron II, bold and italic TG shown) represent the beginning and the end of the introns, respectively.

Table S2. Blast analysis of epimedii *R2R3-MYB* proteins in *Arabidopsis* nr protein database.

Protein name	Amino acid	Top blastp in *Arabidopsis*	Accession number	Score	Query coverage	*E* value
EsMYB1	295	MYB4	NP_195574.1	277	92%	2.00×10^{-74}
EsMYB2	440	MYB61	NP_172425.2	317	99%	2.00×10^{-86}
EsMYB3	308	MYB73	NP_195443.1	249	94%	6.00×10^{-66}
EsMYB4	355	MYB60	NP_172358.1	255	58%	8.00×10^{-68}
EsMYB5	244	MYB3	NP_564176.2	177	60%	3.00×10^{-44}
EsMYB6	366	MYB86	NP_850879.1	249	43%	4.00×10^{-66}
EsMYB7	267	TT2 (MYB123)	NP_198405.1	181	59%	2.00×10^{-45}
EsMYB8	298	MYB	NP_200698.1	295	89%	7.00×10^{-80}
EsMYB9	290	MYB5	NP_187963.1	270	81%	2.00×10^{-72}
EsMYB10	300	TT2 (MYB123)	NP_198405.1	195	55%	7.00×10^{-50}
EsMYB11	266	MYB102	NP_567626.1	177	53%	3.00×10^{-44}
EsMYB12	260	MYB4	NP_195574.1	237	83%	2.00×10^{-62}
EsMYB13	350	MYB44	NP_201531.1	272	97%	1.00×10^{-91}

Table S3. Blast analysis of epimedii *R2R3-MYB* proteins in nr protein database excluding *Arabidopsis*.

Protein name	Amino acid	Top blastp out *Arabidopsis*	Accession number	Score	Query coverage	*E* value
EsMYB1	295	*Gossypium hirsutum*, MYB1	AAA33067.1	302	99%	7.00×10^{-80}
EsMYB2	440	*Betula luminifera*, MYB-like	ACJ38663.1	433	99%	2.00×10^{-119}
EsMYB3	308	*Glycine max*, MYB112	ABH02852.1	292	93%	7.00×10^{-77}
EsMYB4	355	*Vitis vinifera*, MYB60	XP_002271738.1	306	95%	3.00×10^{-81}
EsMYB5	244	*Vitis vinifera*, MybPA2	ACK56131.1	178	78%	1.00×10^{-42}
EsMYB6	366	*Ricinus communis*, MYB	XP_002511336.1	327	99%	2.00×10^{-87}
EsMYB7	267	*Lotus japonicus*, TT2a	BAG12893.1	221	86%	2.00×10^{-55}
EsMYB8	298	*Antirrhinum majus*, DIV	AAL78741.1	358	99%	1.00×10^{-96}
EsMYB9	290	*Vitis vinifera*, MYB5b	AAX51291.1	303	91%	5.00×10^{-80}
EsMYB10	300	*Gossypium hirsutum*, MYB10	AAK19615.1	258	90%	1.00×10^{-66}
EsMYB11	266	*Malus x domestica*, MYB11	AAZ20431.1	181	62%	1.00×10^{-43}
EsMYB12	260	*Vitis vinifera*, MYBC2-L1	ABW34393.1	332	85%	9.00×10^{-89}
EsMYB13	350	*Glycine max*, MYB50	ABH02824.1	358	97%	9.00×10^{-125}

Table S4. List of primers used for conserved domain and the full-length cDNA amplification of epimedii *R2R3-MY*B genes.

Gene name	Degenerate primers for conserved domain (Forward/Reverse) [a]	Gene specific primers for full-length cDNA (Forward/Reverse)	Length of FLC (ORF) [b] (bp)
EsMYB1	5' TGY TGY GAR AAR GCN CAY AYN AA 3'<br>5' TT CCA RTA RTT YTT NAY YTC RTT 3'	5' GACATTTCCCCCAGTTTTCCCT 3'<br>5' AATGATTACAAGTAGGTATTTGAACA 3'	1122 (888)
EsMYB2	5' CAY TCN TGY TGY TWY AAR CAR AA 3'<br>5' CAY AGY TGY TGY TWY AAR CAR AA 3'<br>5' AR RTT YTT DAT YTC RTT RTC NGT 3'	5' GAGACTCTTCTGCTCTTGCTGAT 3'<br>5' GCCTTTATTCAAGCCTTTCAAATT 3'	1460 (1323)
EsMYB3	5' AAR GGN CCN TGG TCN CCN GAR GA 3'<br>5' AAR GGN CCN TGG ASN CCN GAR GA 3'<br>5' AT NGT NGC CCA YTT RTT NCC RAA 3'	5' GCTCACAACCAACCCTCCCCTT 3'<br>5' CTCAAAACCCCAAAACTATCTCCATCA 3'	1085 (927)
EsMYB4	5' AAR GGN CCN TGG ASN CCN GAR GA 3'<br>5' CCA RTA RTT YTT DAT RTC RTT RTC 3'	5' AGCTAGCTGGTGCAAAAGGAATTG 3'<br>5' TGAAGATACCCAAAGGTCCCAGAAC 3'	1297 (1068)
EsMYB5		5' GCAAAACAGAGGGAAAAAAAATAAT 3'<br>5' CTGCAGTTTATTGAAAATTTCCACA 3'	822 (735)
EsMYB6		5' AGATTTGCGTAAAAACCACTTGAT 3'<br>5' CGCAGAATAGAAGAATCCATTTCAC 3'	1563 (1101)
EsMYB7		5' GACAGTAGTCTTTCAAGCCTAAGAAATG 3'<br>5' TACACTGCAGGAACGGAAGACAC 3'	820 (804)
EsMYB8		5' GGGATGGAAATTCAGTCACCAAG 3'<br>5' ATGATTCGGGTCGAATACATAATTGC 3'	1216 (897)
EsMYB9		5' CTGCCGTGCTGTGTAACATAGA 3'<br>5' TCAATGTAACCTATGACAACAAGAAGC 3'	1095 (873)
EsMYB10		5' GGAAATGGGAAGAAGCCCCTGTTGT 3'<br>5' TGATCTATTTACCAAACATTGAATC 3'	986 (903)

Table S4. *Cont.*

Gene name	Degenerate primers for conserved domain (Forward/Reverse) [a]	Gene specific primers for full-length cDNA (Forward/Reverse)	Length of FLC (ORF) [b] (bp)
EsMYB11		5' CAGAGAAAGGAAAAGAAAAATGGTT 3'<br>5' AGAAAAAAAATTTTGATTTTATTTACACC 3'	878 (801)
EsMYB12		5' AGTCTGTCTGTTGGGTGTGGATG 3'<br>5' TCACTGCTTCAGAAATGCTTACCAC 3'	1199 (783)
EsMYB13		5' CCTCCTCTATACAAACCTCCTACCTT 3'<br>5' CGATTTGGCTCTCTGATTTCCTGC 3'	1303 (1053)

[a] two forward degenerate primers of EsMYB2 and EsMYB3 are mixed as the forward primers for conserved domain PCR, respectively. [b] FLC indicates the full-length cDNA, ORF in the bracket indicates the open reading frame.

Table S5. List of primers used for qPCR assay of epimedii *R2R3-MYB* genes.

Gene name	Primers (forward/reverse)	Length of amplicon (bp)
EsMYB1	5' CCTTCCATCATCTTCTTCTTC 3'<br>5' TTCTTCTTCTGTTGCTGTTG 3'	127
EsMYB2	5' AATCCTTCCAACCACAATG 3'<br>5' CTGCTTCCACTATTACTTCTG 3'	144
EsMYB3	5' CCTACTCCTACTGCTGTG 3'<br>5' TGCTTCTCCTTCTCCTTATC 3'	125
EsMYB4	5' AAGTGCGAGGAGTTAGTC 3'<br>5' ATGGTAGGTAGTTTCAGTTG 3'	141
EsMYB5	5' AAAATAATGGGTAGGAAGCC 3'<br>5' AGCAACAGATTCAGCAAG 3'	96
EsMYB6	5' TCATCTCCTTCCTCCATTC 3'<br>5' CCCATTTTACGACGACAG 3'	106
EsMYB7	5' CAGACATCAAGAGAGGAAAC 3'<br>5' CAGCAATCAGAGACCATC 3'	93
EsMYB8	5’ GTCTCTGGAACAATCTGC 3’<br>5’ CCGTAAGGTGGAACAAAC 3’	147
EsMYB9	5' ATACCAACAACCCAAACC 3'<br>5' CTTAGCAATGACTCCAGAAC 3'	77
EsMYB10	5' TGATAGCAGGACGACTTC 3'<br>5' TGGTATAGGTTGTGGTTGG 3'	173
EsMYB11	5' TATGGGCGACTTTGCTAC 3'<br>5' CCTCTCCTCATTTCATCTCC 3'	94
EsMYB12	5' TCCTTCATCTTCATCCTCTTC 3'<br>5' CATCCTTCGCCATTTAGTTG 3'	170
EsMYB13	5' GGTTCGTTTCCTAAGACTG 3'<br>5' GATGAGATGATTGTTGTTGTTG 3'	148
EsActin	5' CACCACAACTGCTGAACG 3'<br>5' AATCGCTCTGCTCCAATG 3'	165

© 2013 by the authors; licensee MDPI, Basel, Switzerland. This article is an open access article distributed under the terms and conditions of the Creative Commons Attribution license (http://creativecommons.org/licenses/by/3.0/).
