# Supplementary material for: Isolation and Molecular Characterization of Thirteen R2R3-MYB Transcription Factors from Epimedium sagittatum
Source: Int J Mol Sci. 2012 Dec 27;14(1):594–610. doi: 10.3390/ijms14010594 (PMC3565284; doi:10.3390/ijms14010594)
Supplement: Supplementary File 2 — Supplemental Figures (PDF, 725 KB) [file ijms-14-00594-s002.pdf]

Supplementary Information

Figure S1. Sequence alignment of epimedii MYB1 and MYB12 proteins with several *R2R3-MYB* proteins containing the C2 repressor motif from other plant species. Identical residues are shown in black and similar residues in gray. The R2 and R3 MYB DNA-binding domains are shown in shaded text. A conserved motif for interaction with bHLH protein is indicated in line. Three conserved motifs, C1, C2 and Zn-finger are indicated in boxes for members of MYB subgroup 4, and of which the Zn-finger motif is located in the EsMYB1 protein sequence, but not in the EsMYB12 protein sequence. Abbreviation: Am, *Antirrhinum majus*; At, *Arabidopsis thaliana*; Br, *Brassica rapa*; Eg, *Eucalyptus gunnii*; Fa, *Fragaria* x *ananassa*; Le, *Lycopersicon esculentum*; Pt, *Pinus taeda*; Ptt, *Populus tremula* x *Populus tremuloides*; Vv, *Vitis vinifera*; Zm, *Zea mays*. GenBank accession numbers for the sequences: AmMYB308, P81393; AtMYB4, NP_195574; AtMYB7, NP_179263; AtMYB32, NP_195225; BrMYB4, ABQ81931; EgMYB1, CAE09058; FaMYB1, AAK84064; LeMYB27, CAA64614; PtMYB14, ABD60279; PttMYB4a, CAD98762; VvMYB4b, ABW34393; ZmMYB31, CAJ42202; ZmMYB42, CAJ42204.

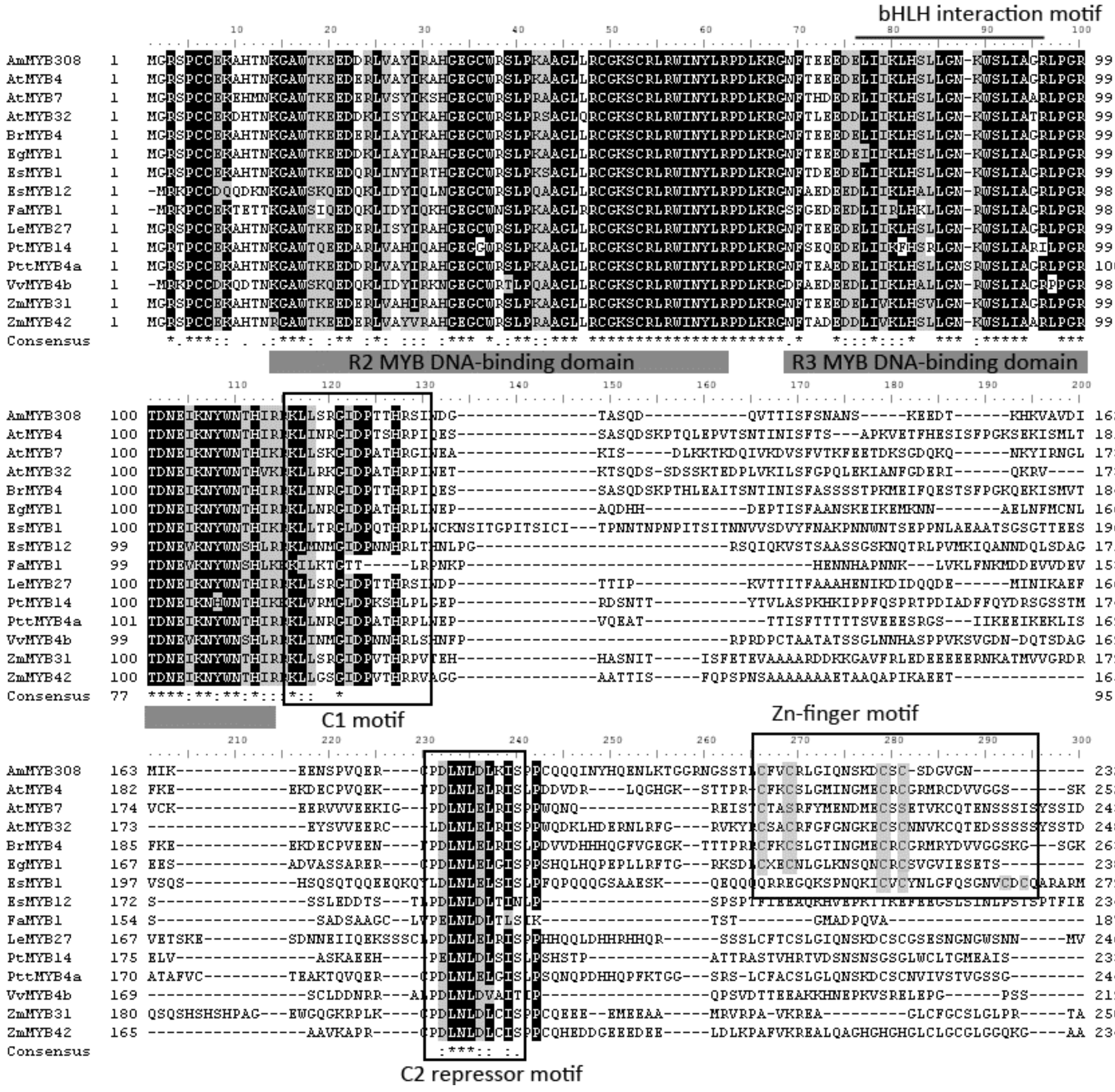

Figure S1. *Cont.*

```
                      310        320        330
                 ....|....|....|....|....|....|.
AmMYB308    232 ------------------------------- 232
AtMYB4      253 GSDMSNGFDFLGLAKKET-TSLLGFRSLEMK 282
AtMYB7      244 ISSSNVGYDFLGLK-----TRILDFRSLEMK 269
AtMYB32     249 ISSS-IGYDFLGLNN----TRVLDFSTLEMK 274
BrMYB4      264 GSDMSNGFDFLGLAKKETNTCLFGFRSLEMK 294
EgMYB1      238 -----VGYDFLGL----KAS-VLDYRS---- 255
EsMYB1      280 VISSDNLHRFLIP---------LDA------ 295
EsMYB12     235 ETQKHVEPKTTKEFEEGSSPTLLLFT----- 260
FaMYB1      187 ------------------------------- 187
LeMYB27     247 SMNIMAGYDFLGL----KTNGLLDYRTLETK 273
PtMYB14     233 ------------------------------- 233
PttMYB4a    245 STSTKNGYDFLGM----KSG-VLDYRSLEMK 270
VvMYB4b     219 --------------------TLLLFG----- 225
ZmMYB31     251 DCKCSS-SSFLGLR-----TAMLDFRSLEMK 275
ZmMYB42     235 GCSCSNGHHFLGLR-----TSVLDFRGLEMK 260
Consensus
```

Figure S2. Sequence alignment of epimedii MYB9 protein with several functionally characterized plant *R2R3-MYB* proteins. Identical residues are shown in black and similar residues in gray. The lines above the alignment indicate the R2 and R3 MYB DNA-binding domains and a conserved motif for interaction with bHLH proteins, respectively. Two conserved motif C1 and C3 are shown in boxes. GenBank accession numbers are as follows (in parenthese): VvMYB5a (AAS68190) and VvMYB5b (AAX51291) from *Vitis vinifera*; AtMYB5 (AAC49311) from *Arabidopsis thaliana*; PH4 (AAY51377) from *Petunia hybrida*; OsMYB4 (BAA23340) from *Oryza sativa*.

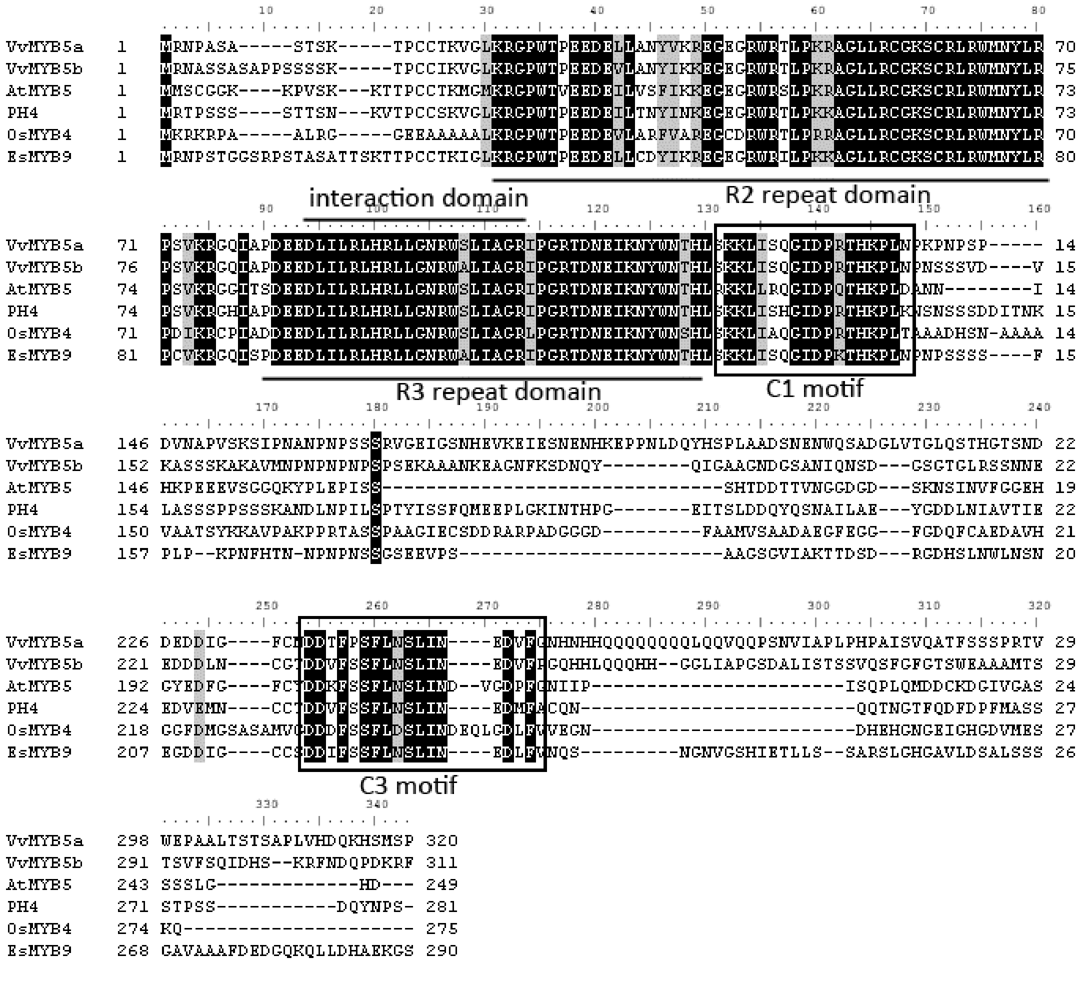

Figure S3. Sequence alignment of epimedii MYB8 with other DIV-like proteins. Identical residues are shown in black and similar residues in gray. The R2 and R3 MYB DNA-binding domains are shown in lines and the "SHAQKY" conserved motif is located in the end of the R3 MYB domain. Another conserved motif is shown in box. Arrowhead indicates the insertion position of intron with the same intron phase in the DIV-like proteins. GenBank accession numbers are as follows (in parenthese): AmDIV (AAL78741) and AmDVL1 (AAL78742) from *Antirrhinum majus*; AtMYB (NP_200698) from *Arabidopsis thaliana*; BlDIV1 (ABN13121) and BlDIV2 (ABN13123) from *Bournea leiophylla*; GmMYB57 (ABH02828) from *Glycine max*; HmDIV1A (ACR09746) and HmDIV2A (ACR09741) from *Heptacodium miconioides*; MtMYB33 (ABR28332) from *Medicago truncatula*; OsMYB (BAD61069) from *Oryza sativa*; ZmDIV (ACG30643) from *Zea mays*.

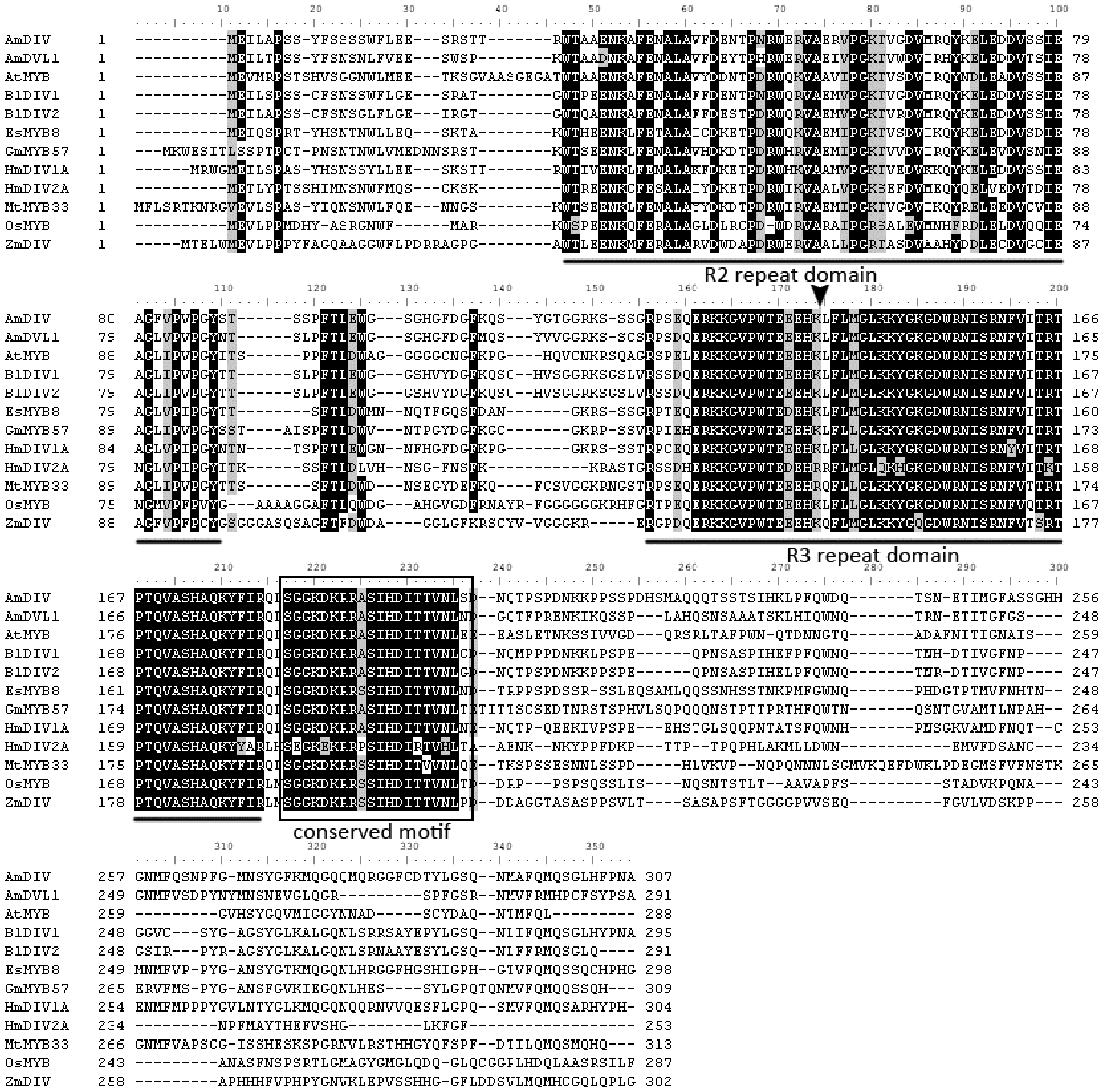

Figure S4. Phylogenetic relationships of thirteen R2R3-MYB proteins from epimedii with other characterized plant *R2R3-MYB* proteins. Topology tree is performed using the neighbor-joining method by the MEGA 4.0 version. The numbers next to the nodes are bootstrap values from 1,000 replicates. Thirteen R2R3-MYB proteins from epimedii are indicated in diamonds. The putative regulatory functions of the different R2R3-MYB proteins in the control of secondary metabolite biosynthesis or other biological processes are indicated. GenBank accession numbers of MYB proteins are shown in the figure, following the gene names.

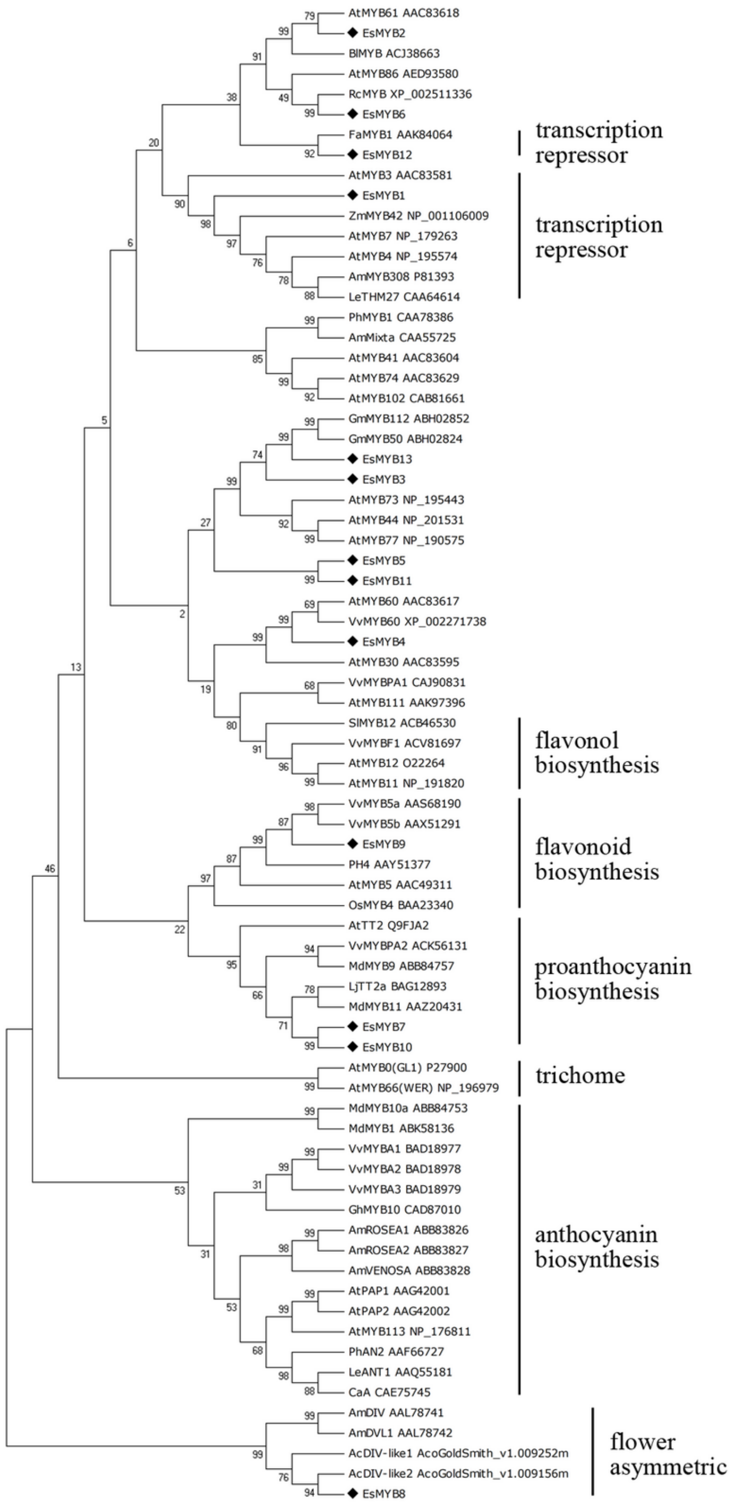

Figure S5. Band size comparison of cDNA and gDNA clones of epimedii *MYB3* and *MYB13* genes. The full-length gene specific primers were used to amplify the cDNA and corresponding genomic DNA (gDNA) clones of *EsMYB3* and *EsMYB13* genes from the cDNA template digested with DNase I and genomic DNA template. The band size is shown equal between the cDNA and gDNA amplification of both *EsMYB3* and *EsMYB13* genes.

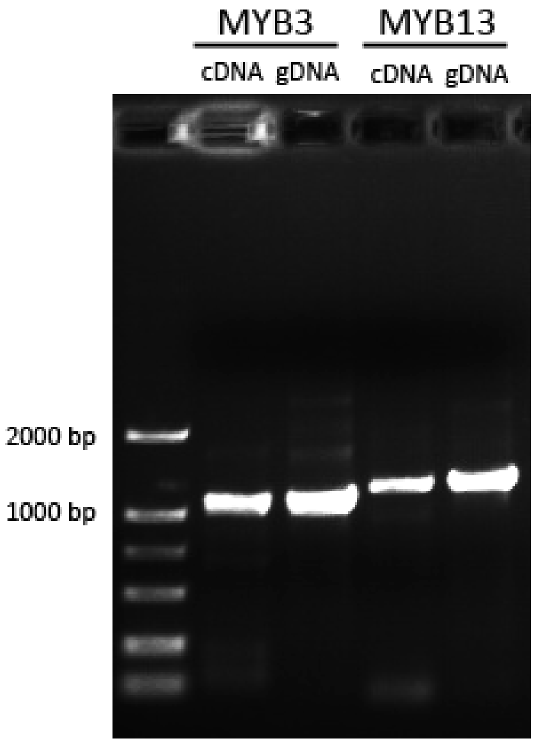

Figure S6. Phenotype observation of transgenic tobacco plants overexpressing *EsMYB9* and empty vector. Overexpression of *EsMYB9* driven by CaMV 35S promoter led to increase the anthocyanin accumulation in flowers, including petals and stamens, compared to the control plants overexpressing the empty vector.

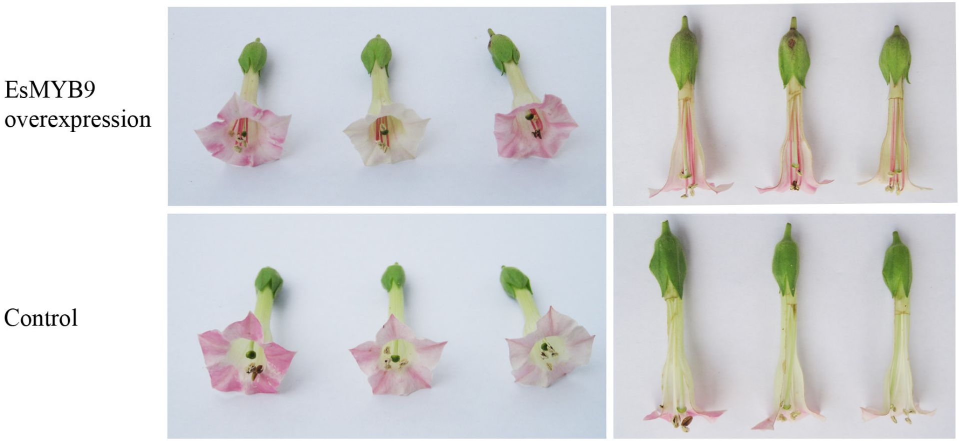

© 2013 by the authors; licensee MDPI, Basel, Switzerland. This article is an open access article distributed under the terms and conditions of the Creative Commons Attribution license (http://creativecommons.org/licenses/by/3.0/).
